# Predicting Fibrosis Progression in Renal Transplant Recipients Using Laser-Based Infrared Spectroscopic Imaging

**DOI:** 10.1038/s41598-017-19006-1

**Published:** 2018-01-12

**Authors:** Vishal K. Varma, Andre Kajdacsy-Balla, Sanjeev Akkina, Suman Setty, Michael J. Walsh

**Affiliations:** 1 Department of Bioengineering, University of Illinois at Chicago, Chicago, USA; 2 Department of Pathology, University of Illinois at Chicago, Chicago, USA; 3 Division of Transplant Nephrology, Department of Medicine, Loyola University Medical Center, Loyola, USA

## Abstract

Renal transplants have not seen a significant improvement in their 10-year graft life. Chronic damage accumulation often leads to interstitial fibrosis and tubular atrophy (IF/TA) and thus graft function loss over time. For this reason, IF/TA has been the chief suspect for a potential prognostic marker for long term outcomes. In this study, we have used infrared spectroscopic (IR) imaging to interrogate the biochemistry of regions of fibrosis from renal transplant biopsies to identify a biochemical signature that can predict rapid progression of fibrosis. IR imaging represents an approach that permits label-free biochemical imaging of human tissues towards identifying novel biomarkers for disease diagnosis or prognosis. Two cohorts were identified as progressors (n = 5, > 50% fibrosis increase between time points) and non-progressors (n = 5, < 5% increase between time points). Each patient had an early time point and late time point biopsy. Collagen associated carbohydrate moieties (*ν*(C–O), 1035 cm^−1^ and *ν*(C–O–C),1079 cm^−1^) spectral ratios demonstrated good separation between the two cohorts (p = 0.001). This was true for late and early time point biopsies suggesting the regions of fibrosis are biochemically altered in cases undergoing progressive fibrosis. Thus, IR imaging can potentially predict rapid progression of fibrosis using histologically normal early time point biopsies.

## Introduction

The kidney is the most commonly transplanted organ in the US^1^. While acute rejection can be controlled by modifying immunosuppressive regimens, chronic allograft injury remains an important cause of graft loss (10 year graft-survival has not improved)^2^. Chronic allograft injury is identified by examining a renal allograft biopsy for vascular and glomerular changes as well as interstitial fibrosis and tubular atrophy (IF/TA)^1^. IF/TA is found in approximately 25% of 1-year post-transplants and leads ultimately to allograft failure^3^.

Fibrosis causes organ damage by a series of cellular and molecular responses to tissue damage^4^. Tissue inflammation often triggers fibrosis from injury to epithelial and endothelial cells and causes further inflammation. While initially beneficial, chronic and progressive fibrotic scarring ultimately leads to organ failure^4^. Fibrogenesis might share common pathways across multiple organs with a general “wounding response” activated by multiple cell/molecular pathways including TGF-β^4^. Physical and biochemical stimulation of myofibroblasts causes extracellular matrix (ECM) secretion and excessive deposition and/or insufficient resorption of ECM is believed to cause fibrosis.

Various disease states have fibrosis as the final common pathway of tissue damage with associated extracellular component deposition, including collagen I, III and IV. Fibrosis is a prognostic indicator of hepatic^5,6^, renal^7,8^ and pulmonary^9^ allograft failure. Studies have focused on genetic/serum markers^3^, or physical property alterations such as tissue stiffness^6^, for predicting fibrosis progression. Recent advances in imaging technology, such as second harmonic generation microscopy for quantification of collagen structure, permit interrogation of fibrosis without losing spatial information^10^. While promising, these methods remain an indirect means to assess the biochemical tissue status.

Identification of biomarkers in regions of fibrosis is an attractive target using spectroscopic techniques^11^. Recent work has demonstrated detect premetastatic changes in the lung microenvironment in response to primary breast tumors^12^. Early diagnosis provides time for intervention as evidenced in liver allografts where progression is determinable early after transplantation^13,14^. IR imaging is a novel approach that permits interrogation of the biochemistry of the fibrotic regions while retaining the spatial information. Recent work in kidney using IR imaging has shown classification of renal fibrosis^15^ and early detection of diabetic nephropathy^16^. IR imaging has shown promise in the renal transplant setting demonstrating the prediction of onset of diabetic nephropathy using early time point biopsies with no histological evidence of diabetic nephropathy^16^.

**Figure 1:**
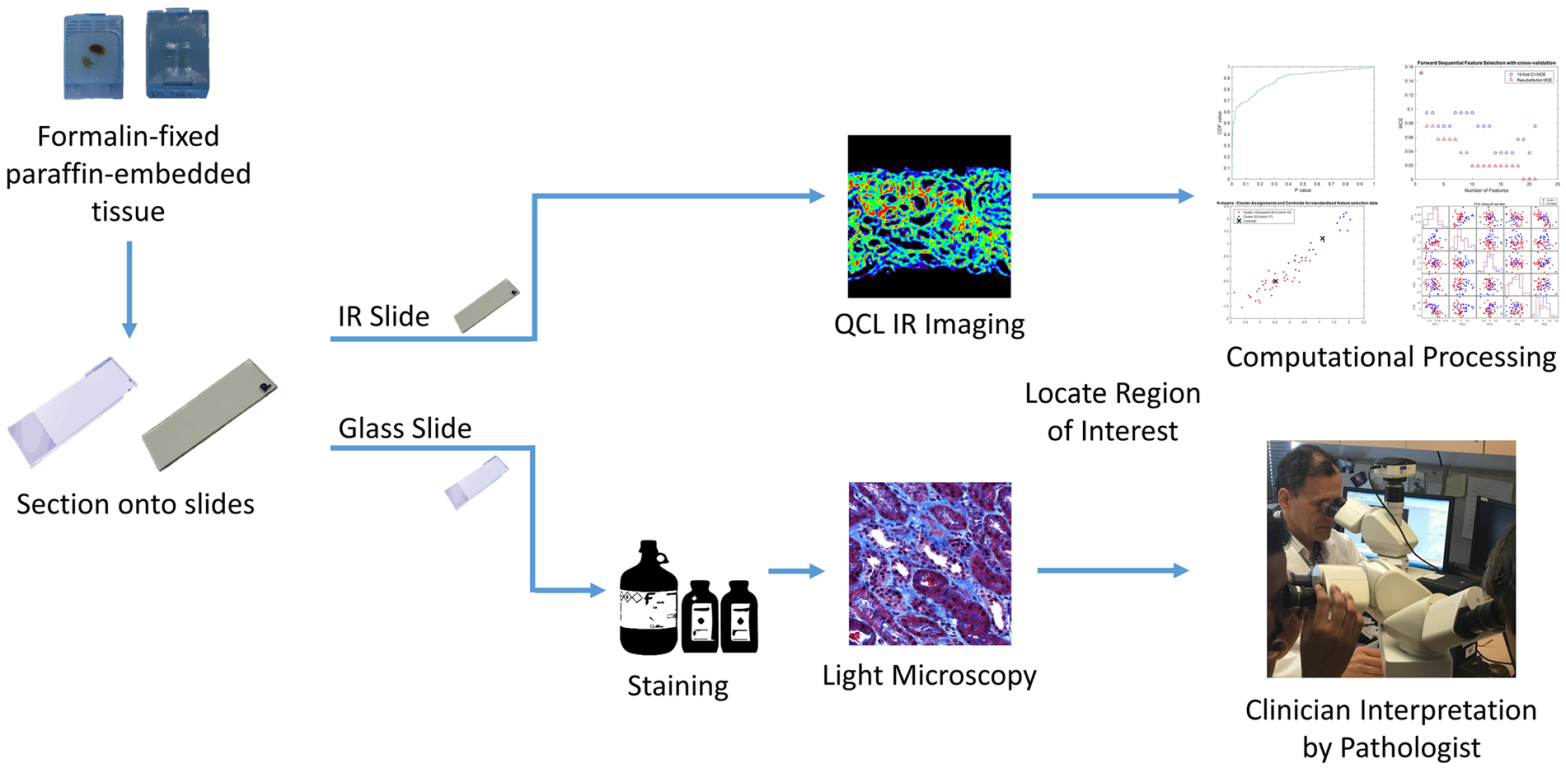
Work flow of Quantum Cascade Lasers (QCL)-based IR imaging systems in respect to histology workflow. QCL systems now allow real time imaging of tissue and discrete frequency data collection. The work flow is very similar to how pathologists currently use stained tissue sections. Once a sample is sectioned on to a compatible substrate, for IR systems, the unstained section can be used for imaging, while for histology the slide must be stained before imaging. The sections can then be visualized in real time in both QCL system and microscopes, and therefore can allow the user to focus on the regions of interest (such as regions of fibrosis). Once a region has been identified, the QCL system then can collect discrete frequency data and using spectral analysis provide the user quantitative information. In traditional histology it is the clinician that interprets the stained sections.

Recent developments in Quantum Cascade Lasers (QCL) enable IR imaging for real-time biopsy imaging and rapid acquisition of biochemical data (Fig. 1) of human tissue^17–19^. Using QCL-IR imaging, it is possible to easily visualize^19^ and biochemically characterize^15^ fibrosis in a label-free manner. As interaction with the IR spectrum is biomolecule specific, we can study biomolecules such as proteins, carbohydrates, lipid, DNA, and collagen content in a sample. Chronic allograft injury as manifested by IF/TA is associated with deposition of various collagens leading to a unique IR signature and providing a novel region to target for potential prognostic biomarkers^20^.

There is currently a lack of tools to predict chronic changes to the kidney post-transplant even when a biopsy has been obtained. There is also a lack of consensus on whether multiple surveillance biopsies in the years following transplantation (protocol biopsies) are of clinical utility when weighed against the risks of performing a renal biopsy^21–23^. The ability to predict patients that will undergo IF/TA and other pathological events that will ultimately lead to deterioration of graft function would be very useful. Determining ‘at-risk’ patients could allow for more tailored use of protocol biopsies^22^. In addition, knowing which patients will undergo fibrosis will allow for developing better clinical trials to test novel anti-fibrotic drugs. Furthermore, as new immunosuppressive regimens are developed there will be an increased focus on creating individually tailored treatments^21^.

## Results

**Figure 2:**
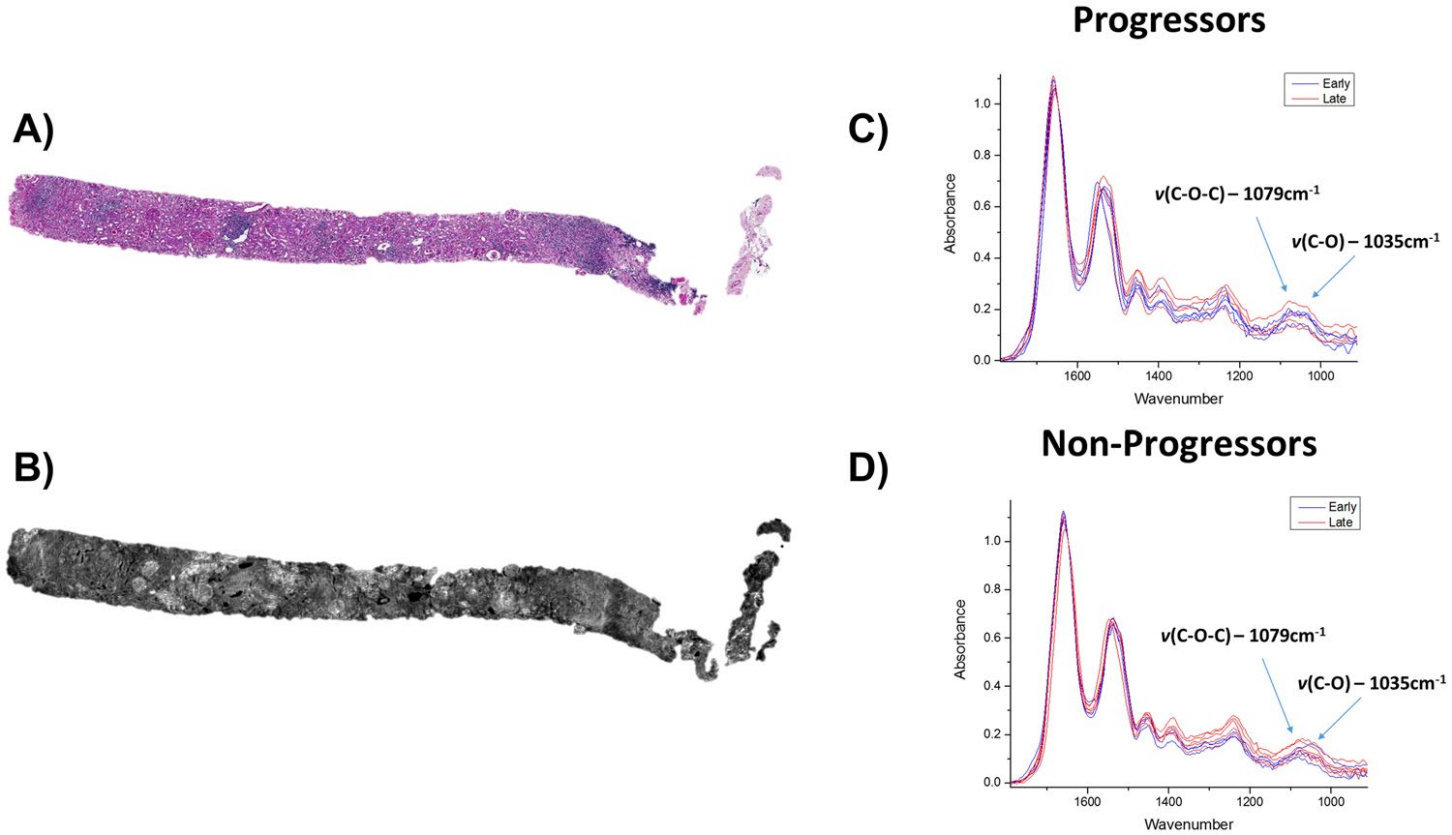
Real time chemical imaging allows for rapid data collection. Two serial sections were obtained on glass slide and infrared (IR) compatible substrate. One section was stained with Period Acid Schiff (**A**) while the other unstained section was imaged using QCL-based IR imaging (**B**). When region of interest is located (either via using a serial section stained tissue or real time imaging), the biochemical data can then be collected. This was done for Progressors (**C**) and Non-Progressors (**D**) cohorts for both early and late time point biopsies. While in this case the region of 1800–900 cm^-1^ was collected, it is also possible to just collect spectral frequencies of interest such as *v*(C-O-C) and *v*(C-O) which have been identified as spectral collagen markers.

In order to identify biochemical signatures of fibrosis, a pathologist graded Masson’s trichrome stained renal allograft biopsy sections using the Banff 2005 classification for IF/TA across two biopsy time points. The Banff 2005 classification determined that chronic allograft injury with fibrosis can have a multifactorial etiology with vascular and tubular changes also present^24^. In this study we have restricted our focus to extracting IR signatures from the regions of fibrosis. To ensure detection of only signatures related to fibrosis progression and minimize influence from any confounding pathological factors, very strict selection criteria of patient samples were used. Patients selected had to be non-diabetic and have had two biopsies within the first two years after transplantation. Patients were restricted to biopsies being within the first two years post-transplant to ensure other chronic conditions associated with transplantation were not being measured. In addition, the patients could not demonstrate any clinical complications such as infection or evidence of rejection. While this limited the number of patients eligible for the study it ensured confidence in the detected signatures by having a very well-defined patient cohort. Using the IF/TA scores, these candidates were grouped into two categories based on fibrosis progression: progressors (n = 5) and non-progressors (n = 5) (patient details in Supplemental Table 1). Serial sections of formalin-fixed paraffin-embedded (FFPE) renal biopsies were sectioned on to glass slides and used for Periodic Acid Schiff (PAS, Fig. 2A) and Masson trichrome staining while an additional section was placed on to a barium fluoride slide for QCL-IR imaging (Fig. 2B). To accurately quantify the interstitial fibrosis, image analysis was performed on the digitally scanned Masson trichrome stained slides for a percent fibrosis score (Supplemental Fig. 1).

Each cohort contains an early and a late time-point biopsy for each subject (2 biopsies total per patient). A patient was classified as being a progressor if there was an increase in fibrosis of more than 50% between time-points (3–6 months post-transplant for early, and 6–12 months for the late time points) and classified as a non-progressor if there was less than 5% increase in fibrosis (0–24 months post-transplant for early, and 6–36 months for the late time points) (Supplemental Table 1). We excluded patients that had a percentage increase of fibrosis between 5 and 50%. This was due to fact that tissue biopsies only sample a small portion of the kidney and thus there is a risk that you are observing differences in sampling of fibrosis rather than true fibrosis progression. As such by only using patients with over 50% fibrosis ensured that we could be confident that our progressive cohort was truly progressive.

Using the stained tissue sections (Fig. 2A) as a guide, we scanned regions of fibrosis from each biopsy (Fig. 2B) using QCL-IR imaging. The high spatial resolution of QCL-IR imaging permits extraction of biochemical data specifically from the interstitium, which was then averaged from multiple pixels in the IR image per patient and compared at each time point (Fig. 2C,D). The spectral fingerprint region (1800–900 cm^−1^) is well known for yielding unique collagen-associated spectral signatures^20^ of the *ν*(C–O) and *ν*(C–O–C) carbohydrate moieties (which exhibit absorption at 1035 cm^−1^ and 1079 cm^−1^ respectively). As shown in previous work^14^, this region contains information associated with diabetic nephropathy (1080 cm^−1^ and 1030 cm^−1^). Therefore, only non-diabetic cases were selected for this study to exclude confounding factors associated with diabetic nephropathy in this region. In this fashion, the spectral signatures of collagen I, III and IV were obtained.

There is poor correlation (Pearson correlation coefficient, r = 0.32) between IF/TA grades/percent fibrosis using a simple spectral peak ratio of 1079 cm^−1^: 1035 cm^−1^ (Supplemental Fig. 2), but there is a clear cluster separation of the progressor and non-progressor groups using this spectral ratio. This suggests that the biochemical changes observed in the fibrosis are not simply due to the overall percentage of tissue fibrosis.

**Figure 3:**
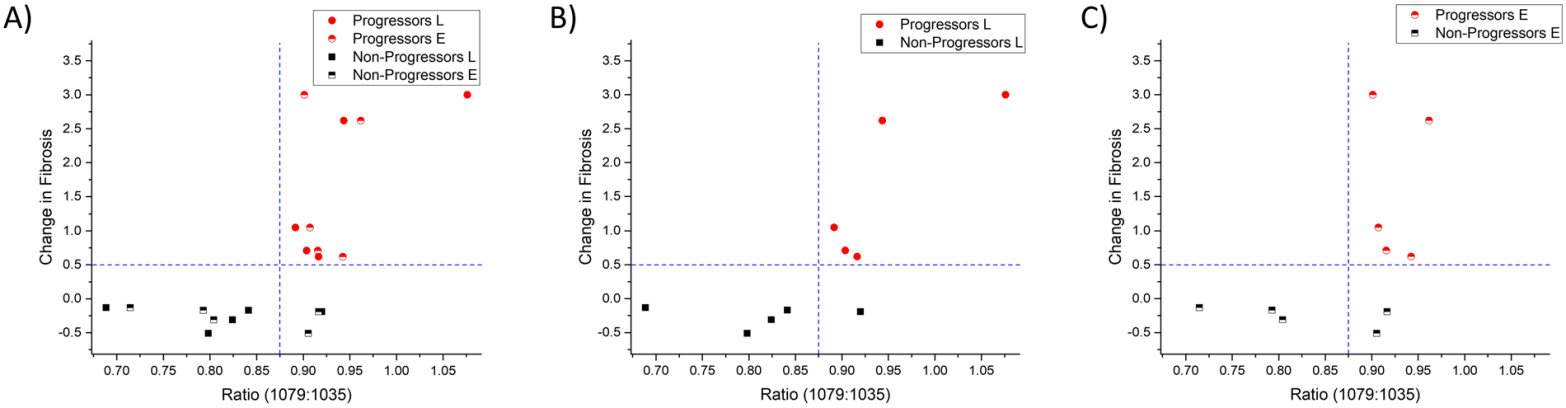
Change in interstitial fibrosis and tubular atrophy (IF/TA) plotted against spectral ratio of v(C-O-C) and v(C-O) (1035 and 1079 cm-1 respectively). Each biopsy had IF/TA quantified using image analysis software (Histolab) which was then used to compute the change in fibrosis between the early and late time point biopsies. The spectral ratio was extracted from the regions of fibrosis in the biopsies. The data showed good correlation between the change in fibrosis and spectral ratio (r = 0.65) for all times points (**A**). Late (**B**) and Early (**B**) time points also showed discrimination using the spectra ratio.

Next, we plotted the change in fibrosis between time points for each patient against the 1079 cm^−1^: 1035 cm^−1^ spectral ratio (Fig. 3A–C). Good correlation (Pearson correlation coefficient, r = 0.65) is found using just this spectral ratio for percentage change in fibrosis. Both early (Fig. 3C) and late (Fig. 3B) time point biopsies for the progressors cohort showed clear separation, suggesting that the fibrotic region of progressors vs. non-progressors are biochemically different between these two groups. Thus, even the early time point biopsies (Fig. 3C) contain biochemical information that may help identify a patient as a progressor using a 3 to 6 month biopsy that otherwise appears histologically normal. The differences in the spectral biomarkers were statistically significant between progressors and non-progressors (p = 0.001).

**Figure 4:**
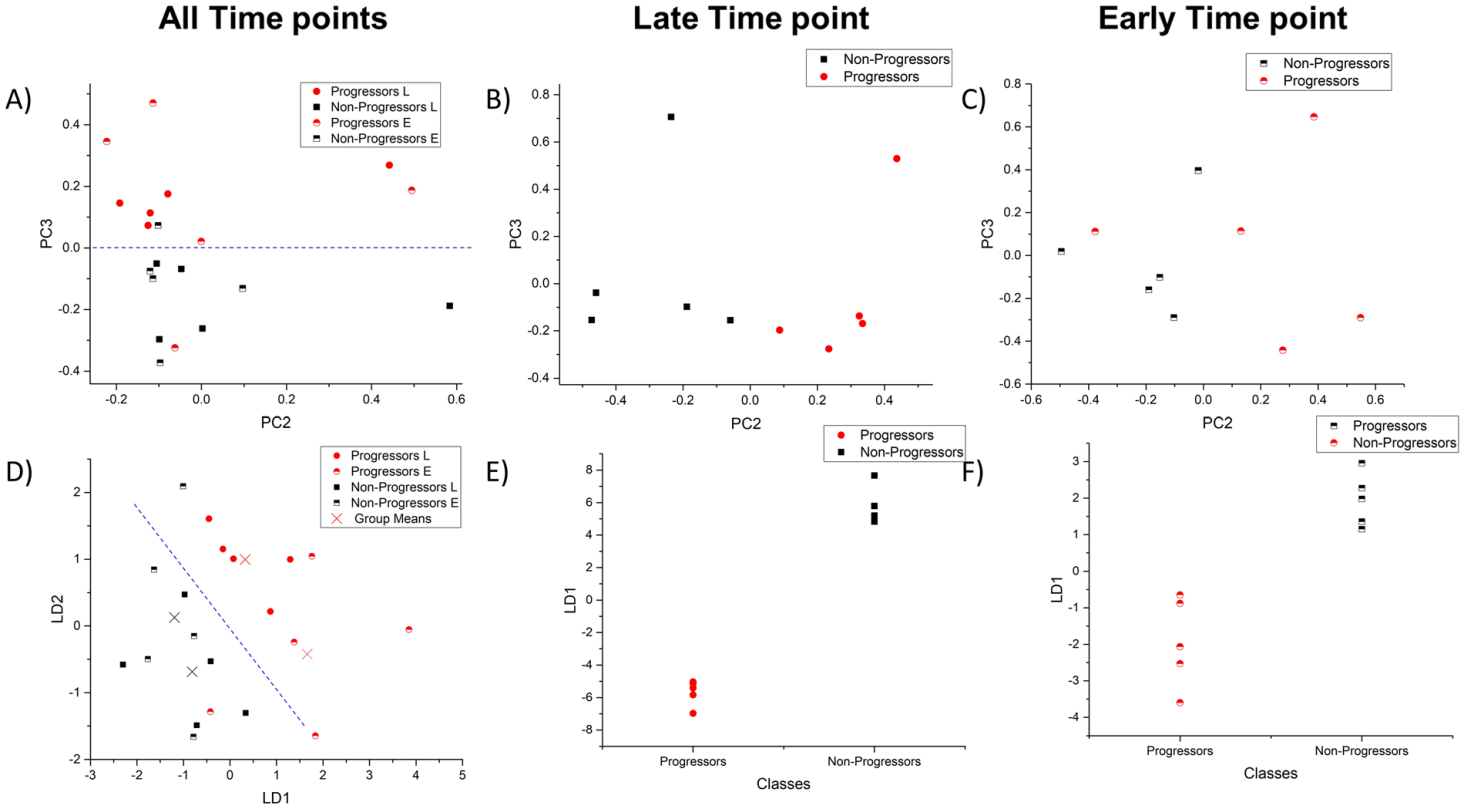
Multivariate time point analysis of biopsies for classification of progressors. Using Principal Component Analysis (PCA) to reduce the data dimensionality (using the 1800–900 cm^−1^) while maintaining the variance in an unsupervised fashion, it was possible to see segregation between all-time points (**A**–**C**). This shows alteration in the inherent biochemistry of fibrosis between the progressors and non-progressions. Furthermore, if Linear Discrimination Analysis is used to classify the reduced dataset, it is possible to see near perfect segregation between progressors and non-progressors at all time points (**D**–**F**).

Finally, we expanded our analysis beyond two IR spectral frequencies and applied the multivariate data analysis technique, principal component analysis (PCA). PCA helps identify sources of spectral variance in large multi-dimensional datasets and permit visualization of clustering in an unsupervised fashion. PCA can be further coupled with Linear Discriminant Analysis (LDA) to identify sources of inter-group variance and maximize discrimination between groups which allows for analysis of clustering based on biochemical similarity. The Principal Components were carefully selected to avoid overfitting in the LDA^25^. Using either PCA (Fig. 4A–C) or PCA-LDA (Fig. 4D–F), it was possible to see distinct separation between progressors and non-progressors. This suggests that there are inherent biochemical differences between the two cohorts for both late (Fig. 4B) and early (Fig. 4C) time point biopsies. Interestingly, there appeared to be very little separation between the early and late biopsies of the progressors. This suggests that the biochemistry of the fibrosis in progressors is different from non-progressors at an early stage and thus that the trajectory of the patient of being at risk or sensitized to fibrosis progression occurs soon after transplantation. It has been previously discussed that the first few months after transplantation are important in the future development of chronic transplant injury^21^.

## Discussion

QCL-IR imaging allows for real-time imaging and collecting data only from the spectral regions of interest from unstained tissue sections. This allows us to quickly find regions of interest (such as fibrosis) and collect only the spectral frequencies of interest (such as 1079 cm^−1^ and 1035 cm^−1^) in a rapid manner. We have shown that the fibrosis is biochemically different in patients undergoing rapid progressive fibrosis compared to non-progressors. Biochemical differences from the fibrosis of the two groups is identifiable in both normal appearing early time point biopsies (3 to 6 months post-transplant) as well as late time point biopsy. This shows great promise of identifying patients soon after transplantation with histologically normal biopsies that are at risk of undergoing rapid fibrosis progression. There are currently no tests that can predict those patients who will undergo rapid progression of fibrosis. This could ultimately allow for earlier clinical intervention than currently possible or determining the intensity of surveillance of patients. For example, some institutions will obtain protocol biopsies every few months post-transplant however due to the dangers associated with obtaining a biopsy there is a movement to reducing biopsies. If a patient was flagged as high risk they could be biopsied at increased intervals. IR imaging may also be a valuable tool in assessing novel fibrotic treatments on patients, specifically treatments that target reversal of scarring or prevention of further scarring of tissue^26^. It is currently difficult to assess anti-fibrotic drug efficacy in renal transplant patients as it is unknown which of the patients would have undergone fibrosis and thus benefited from test drugs. Future work will focus on increasing sample sizes and exploring the different etiologies that can cause IF/TA. In addition, we will further explore the precise biomolecular components that are being altered in the regions of fibrosis. IR imaging may be a very useful tool that will allow for better predictive capabilities about recurrence of diabetic nephropathy^16^ and rapid progression of IF/TA in renal transplant recipients and help clinicians maximize graft life.

## Methods

Protocol biopsies performed on non-diabetic individuals without any evidence of rejection or viral infections were included in the study. The Office for the Protection of Research Subjects at University of Illinois at Chicago approved all experimental protocols (IRB#2016–0581). All methods were carried out in according to relevant guidelines and regulations. Waiver of informed consent was obtained for this research under 45 CFR 46.116 (d). The renal biopsy FFPE tissue blocks were obtained from the University of Illinois at Chicago, Tissue Bank. Serial sections were acquired on barium fluoride substrate for IR and glass slides for Periodic acid Schiff (PAS) and Masson’s trichrome staining. The Masson’s trichrome and PAS stained slides were scanned using Aperio ScanScope CS (Leica Biosystems, Nussloch, Germany) and analyzed using image analysis software Histolab (Gothenburg, Sweden). IR images were obtained with Spero QCL System (Daylight Solutions, San Diego, CA) in transmission mode using 12.5 × focusing objective (NA = 0.70) with a pixel size of 1.4 × 1.4 µm. Data was collected over the range 1800cm^−1^– 900 cm^−1^ using 4 cm^−1^ step size. The data was processed in ENVI-IDL (Harris Geospatial Solutions, Broomfield, CO) for standard linear baselining. Multivariate analysis was performed in MATLAB 2016a (MathWorks, Natick, MA) and figures were created using Origin Pro 9.0 (OriginLab, Northampton, MA).

### Data Availability

The datasets generated during and/or analyzed during the current study are available from the corresponding author on reasonable request.

## Electronic supplementary material

Supplementary Data
