# Supplementary material for: Predicting Fibrosis Progression in Renal Transplant Recipients Using Laser-Based Infrared Spectroscopic Imaging
Source: Sci Rep. 2018 Jan 12;8:686. doi: 10.1038/s41598-017-19006-1 (PMC5766495; doi:10.1038/s41598-017-19006-1)

Predicting Fibrosis Progression in Renal Transplant Recipients Using Laser-Based Infrared Spectroscopic Imaging

Supplemental Data

Vishal K. Varma, Andre Kajdacsy-Balla, Sanjeev Akkina, Suman Setty, and Michael J. Walsh.

Supplemental Table 1. Table of patient clinical values obtained at the time of biopsy. The table includes time of biopsy since transplantation, serum creatinine in mg/dl (Creat), creatinine clearance in ml/min (CrCl), glomerular filtration rate in ml/min (GFR), total urine albumin mg/gm creatinine, total urine protein gm/gm creatinine, chronic glomerular score (cg), chronic tubular score (ct), chronic interstitial score (ci), and chronic tubular + chronic interstitial score (cti).

Patient No.	Cohort	Time (months)	Creat	CrCl	GFR	Albumin	Protein	cg	ct	ci	cti
**1**	Progressors	12	1.60	26.90	42.40	13.00	0.09	2	2	3	5
**1**	Progressors	6	1.20	36.30	59.30		0.04	1	1	1	2
**2**	Progressors	12	1.15	69.40	76.00			0	1	1	2
**2**	Progressors	6	1.20	69.40	69.30			1	0	0	0
**3**	Progressors	6	1.50	75.90	61.10	42.00	0.71	0	1	1	2
**3**	Progressors	3	1.70	67.00	52.90			0	0	1	1
**4**	Progressors	12	1.60	53.40	49.50	27.00		0	0	1	1
**4**	Progressors	3	1.80	36.30	43.40	59.00		1	1	0	1
**5**	Progressors	12	1.50	69.60	56.00		0.20	0	1	1	2
**5**	Progressors	6	1.30	80.30	66.00	62.00	0.28	1	0	1	1
**6**	Non-Progressors	6	1.60	54.80	56.80			0	0	0	0
**6**	Non-Progressors	0	2.00	43.80	43.90		0.37	0	1	1	2
**7**	Non-Progressors	36	1.80	62.60	43.80	1.00		0	1	0	1
**7**	Non-Progressors	24	1.70	65.10	47.00	2.00		0	1	0	1
**8**	Non-Progressors	12	1.10	42.10	72.30	12.00	0.17	0	1	1	2
**8**	Non-Progressors	3	1.30	35.60	59.60	3.00	0.13	0	0	1	1
**9**	Non-Progressors	6	1.56	69.10	66.10	82.00	0.20	0	0	0	0
**9**	Non-Progressors	3	1.40	76.80	74.80	34.00		0	0	1	1
**10**	Non-Progressors	12	1.50	47.80	61.60	36.00	0.20	1	1	2	3
**10**	Non-Progressors	6	1.60	44.90	57.20	3.00	0.07	2	1	3	4

Supplemental Figure 1. Extracting of Morphological data using adjacent sections. Histolab was used to quantify the amount of fibrosis in the biopsies as shown below. Each field was taken at 20X, and analyzed independently. At the end, all of the fibrosis was summed and then divided by the area of the biopsy to give a percent fibrosis.

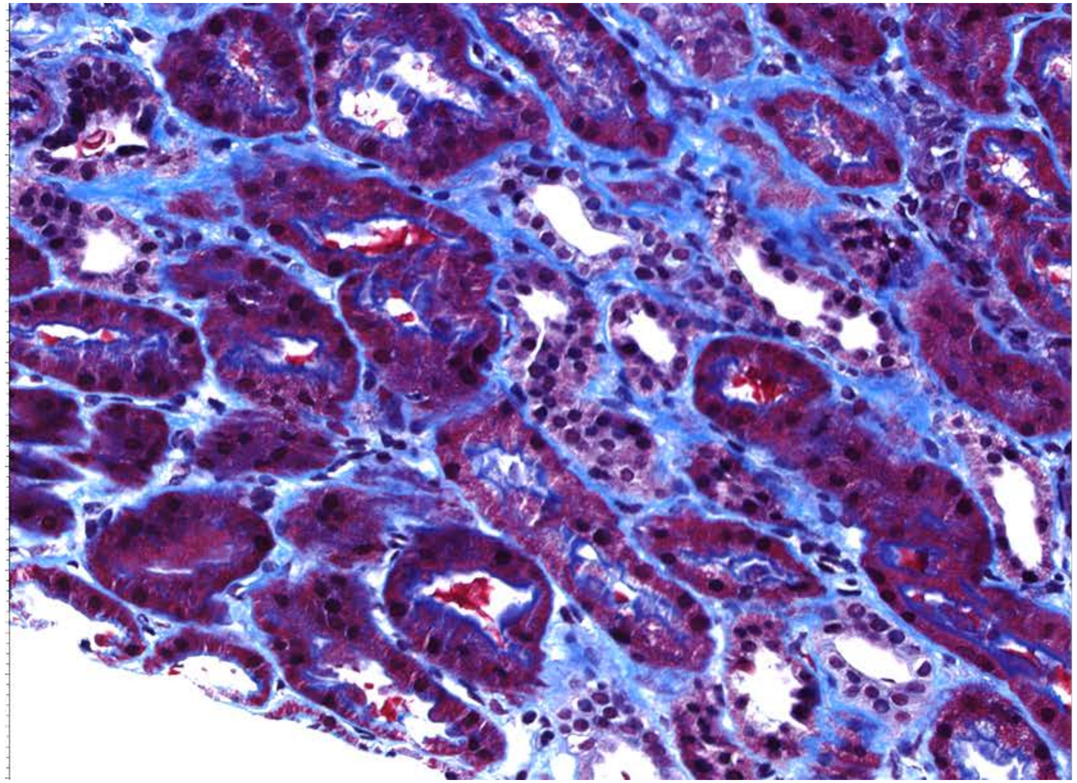

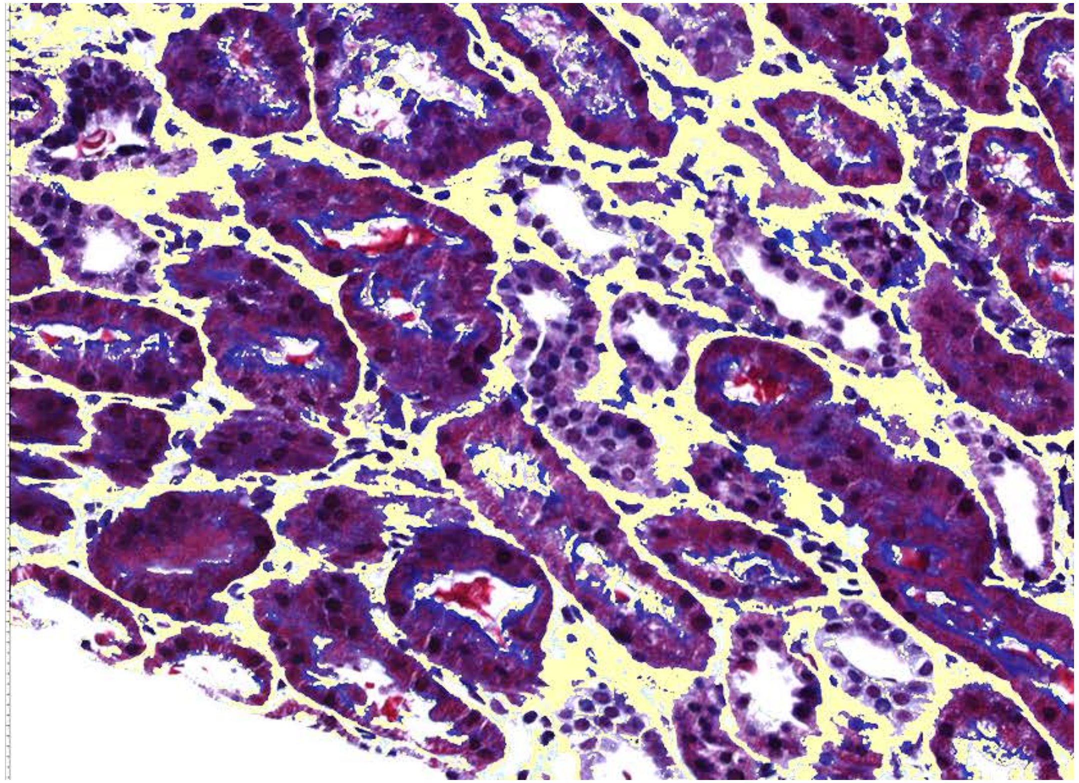

Supplemental Figure 2. Fibrosis percent vs spectral biomarker. Since fibrosis percent is not a good measurement to distinguish between progressors and non-progressors, it is important to show that our data does not correlate with the amount of fibrosis.

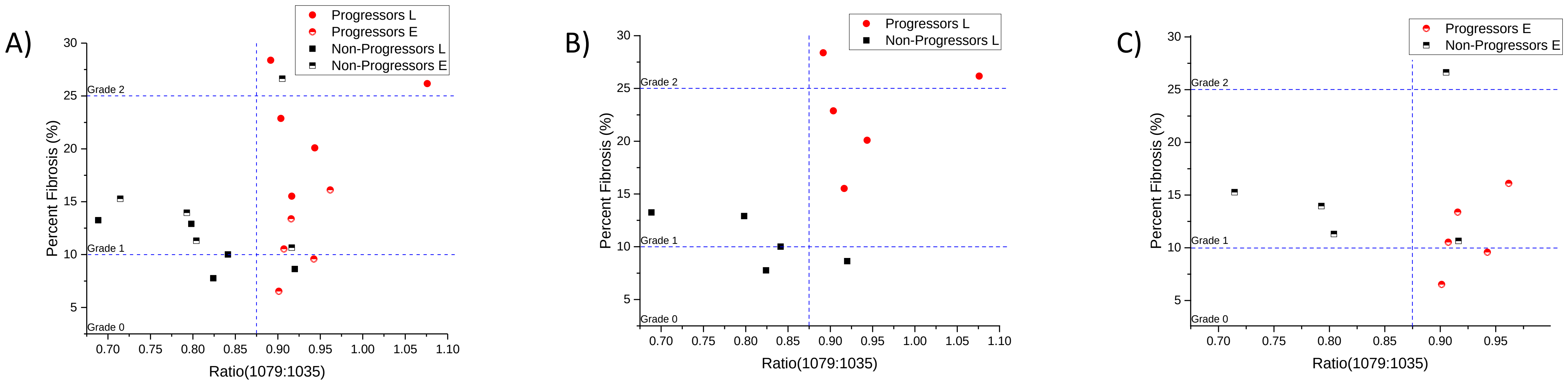

Supplement: Supplementary file 1 — Supplementary Data [file 41598_2017_19006_MOESM1_ESM.pdf]
